# Distribution of Refractive Errors among Healthy Infants and Young Children between the Age of 6 to 36 Months in Kuala Lumpur, Malaysia—A Pilot Study

**DOI:** 10.3390/ijerph16234730

**Published:** 2019-11-27

**Authors:** Arifah Nur Yahya, Sharanjeet Sharanjeet-Kaur, Saadah Mohamed Akhir

**Affiliations:** 1 Optometry & Vision Science Programme, Faculty of Health Sciences, Universiti Kebangsaan Malaysia, Jalan Raja Muda Abdul Aziz, Kuala Lumpur 50200, Malaysia; arifahnur@yahoo.com (A.N.Y.); drsaadah@ukm.edu.my (S.M.A.); 2 Ophthalmology Department, Hospital Queen Elizabeth, Karung Berkunci No. 2029, Kota Kinabalu 88586, Sabah, Malaysia

**Keywords:** infants, young children, refractive error

## Abstract

Uncorrected refractive error, especially myopia, in young children can cause permanent visual impairment in later life. However, data on the normative development of refractive error in this age group is limited, especially in Malaysia. The aim of this study was to determine the distribution of refractive error in a sample of infants and young children between the ages of 6 to 36 months in a prospective, cross-sectional study. Cycloplegic retinoscopy was conducted on both eyes of 151 children of mean age 18.09 ± 7.95 months. Mean spherical equivalent refractive error for the right and left eyes was +0.85 ± 0.97D and +0.86 ± 0.98D, respectively. The highest prevalence of refractive error was astigmatism (26%), followed by hyperopia (12.7%), myopia (1.3%) and anisometropia (0.7%). There was a reduction of hyperopic refractive error with increasing age. Myopia was seen to emerge at age 24 months. In conclusion, the prevalence of astigmatism and hyperopia in infants and young children was high, but that of myopia and anisometropia was low. There was a significant reduction in hyperopic refractive error towards emmetropia with increasing age. It is recommended that vision screening be conducted early to correct significant refractive error that may cause disruption to clear vision.

## 1. Introduction

Uncorrected refractive error is one of the leading causes of visual impairment in the world. Over the years, a number of investigators have performed cross-sectional and longitudinal studies on the refractive status of neonates, infants, toddlers, preschool and school-aged children. However, more population-based studies on the prevalence of refractive error have been conducted in school-aged children as compared to infants and toddlers. A recent meta-analysis by Hashemi et al. [1] has estimated pooled prevalence (EPP) globally and regionally as defined by the World Health Organization. For children (defined as less than 20 years of age), the estimated pooled prevalence of astigmatism (defined as >0.50 D) was 14.9% (95% confidence interval 12.7–17.1), myopia (defined as ≤ −0.50 D) was 11.7% (95% confidence interval 10.5−13.0) and hyperopia (defined as ≥ +2 D) was 4.6% (95% confidence interval 3.9−5.2). The EPP of myopia, hyperopia and astigmatism in the WHO regions are shown in Table 1. The study also found that the prevalence of refractive errors varied between countries, suggesting there may be genetic and /or environmental influences. There was also a notable increase in the prevalence of myopia from 10.4% in 1993 to 34.2% in 2016. However, this meta-analysis was limited because it included studies which did not perform cycloplegic refraction and the age range of the subjects categorized as children was very large—that is, between 5 years to 18 years.

In infants, full term babies commonly reveal high levels of hyperopia and astigmatism which later reduces rapidly during the first year of life [2]. The process that occurs during the normal growth period by which the eye changes from a state of ametropia, regardless of whether it is initially hyperopia, myopia, or astigmatism, to low hyperopia or emmetropia, is known as emmetropization, which is completed in 82% of full-term infants by 12 months [3]. However, the refractive state may eventually develop towards a different refractive status other than emmetropia. Previous studies have also shown that hyperopia tends to decrease with age [4,5,6]. Infants are typically born with relatively steep corneas resulting in relatively high astigmatism, generally against-the-rule. Astigmatism then decreases rapidly until, approximately, the age of 18 years, and with-the-rule astigmatism is the more typical pattern [7,8].

Many studies have documented ethnic-related differences in the prevalence and magnitude of astigmatism in children. Most studies have shown that there is a prevalence of high astigmatism in Native American [9,10,11], East Asian [12], and Hispanic [13,14] populations. Wen et al. [6] showed similar trends in the prevalence of high astigmatism (>1.5 D) in Hispanics (6.8%), Asians (8.29%), African-Americans (6.6%), and non-Hispanic whites (6.33%) after adjusting for age and gender. The increased prevalence of astigmatism in these populations was suggested to be due to genetic factors and/or eyelid pressure [8].

Therefore, there are both age- and ethnic-related differences in the prevalence of different types of refractive errors. Knowing the refractive error that is within the normal range for the child’s age helps when considering prescribing glasses for a young child [15]. Uncorrected significant refractive error in childhood can be associated with the development of amblyopia and strabismus, resulting in permanent vision loss that can affect the child’s capability and later academic performance. Amblyopia affects 1–5% of populations [16]. Ingram and Barr [17] (1979) reported that the incidence of amblyopia at 3.5 years old is 48% if the child is having ≥ +3.50 D of refractive error at 1-year old age. The result is consistent with a later study which revealed the abnormal level of hyperopia (≥ +3.50 D) in infants being associated with a higher risk of amblyopia (37.5%) and strabismus (21%) at four years of age [18].

In our present cross-sectional study, we aim to describe the distribution of refractive error in a sample of healthy, full term infants and young children aged 6 to 36 months in Kuala Lumpur and to determine the association between refractive error group with age, gender and race.

## 2. Materials and Methods

### 2.1. Sampling

This was a prospective, cross-sectional study with convenience sampling among infants and young children who attended a government health clinic in Sentul, Kuala Lumpur. The inclusion criteria were Malaysian healthy infants and children aged 6 months to 36 months, both gender, normal gestation of ≥37 weeks, normal or uncomplicated caesarean section delivery and birth weight of ≥2500 g. Children with fever, having a history of cardiac, liver, asthma, other respiratory diseases, ocular disease or active ocular inflammation, cataract, glaucoma, disc anomaly or squint were excluded.

This study was conducted according to the tenets of Declaration of Helsinki of human subjects, and research approval was obtained from the Research Ethics Committee, Universiti Kebangsaan Malaysia (NN-070-2015) and Medical Research and Ethics Committee (MREC) Ministry of Health Malaysia (NMRR-15-1250-26657). Written informed consent was obtained from the parents/guardians after a detailed explanation of the study.

### 2.2. Sample Size Calculation

The sample size calculation was based on the prevalence of myopia of 11% in a study of Prevalence of Refractive error in Singaporean Chinese Children: The Strabismus, Amblyopia and Refractive Error in Young Singaporean (STARS) Study [4]. The calculated sample size was, as follows.

The formula for single proportion (Cochran 1963) [19]:

(1) $$n=\frac{Z^{2}P(1-P)}{d^{2}}.$$

*n* = sample size.

*Z* = statistic for the level of confidence. For level of confidence of 95%, Z value is 1.96.

*P* = 0.11 (expected prevalence based on study done by Dirani et al. (2010))

*d* = precision = 0.05

$n=\frac{{\left(1.96\right)}^{2}(0.11)(1-0.11)}{{\left(0.05\right)}^{2}}=150$

### 2.3. Refractive Error Screening Procedure

The eye and vision screening for data collection was carried out in a period of two months. All children with an age range between 6 months to 36 months who attended Sentul Health Clinic, including children who had been scheduled for immunization, were screened. Parents or guardian of children who fulfilled the eligibility criteria were given a verbal explanation about the study, as well as the Patient Information Sheet. Written informed consent was obtained from the child’s parents or guardian if they agreed to participate in this study.

Refractive errors were measured in 151 healthy subjects. Cycloplegic refraction was performed by the same, well-trained optometrist. Prior to installation of cycloplegia, anterior chamber depth shadow test was done to ascertain if eye dilating drops were safe to use for the child. Cyclopentolate 1% (2 drops with 5 min interval each drop) was used. The refraction was conducted at least 30 min after instillation of the second drop of Cyclopentolate 1%. The effect of the drops and the nature of the eye assessment were explained to all parents or guardian. Children with a significant refractive error were given a prescription for spectacles.

### 2.4. Definition of Refractive Error

Refractive error was recorded as spherical equivalent (SE), which was defined as the spherical power plus half of the negative cylinder in dioptre (D) unit. Myopia was defined as SE refractive error at least −1.00 D, and hyperopia as SE refractive error of at least +2.00 D. Astigmatism was defined as a cylindrical measurement (negative notations) of at least 1.50 D. Axis of the cylinder was categorized as with the rule (minus cylinder axis at 180° ± 15°), against the rule (minus cylinder axis at 90° ± 15°), or oblique (all else). Anisometropia was defined as a difference of at least 2.00 D between eyes in SE or cylinder. Refractive error was defined as SE ≥ +2.00 D or ≥ −1.00 D. This definition was also used to determine refractive error groups; with refractive error having SE ≥ +2.00 D or ≥ −1.00 D and without refractive error having SE between +1.75 D to −0.75 D.

### 2.5. Statistical Analyses

Normality of the data was tested using the Kolmogorov-Smirnov test (large sample size). Spearman correlation of mean spherical equivalent (SE) and mean cylindrical power of right and left eyes were determined. The data were analysed using SPSS version 20.0 (IBM, Armonk, NY, USA). The distribution of refractive error was reported in a descriptive method. The mean, standard deviation, 95% confidence interval (CI), median and range were used to illustrate the distribution of the data. 95% CI for proportion was conducted using the Clopper-Pearson “exact” method. Spearman correlation between SE and age was performed. Chi-square was used to determine the association between refractive error group with race and gender. The refractive error groups were with refractive error and without refractive error.

## 3. Results

The study population consisted of 151 infants and young children with a mean age of 18.09 ± 7.95 months (age range 6 months to 36 months). Seventy-eight percent (78%) of the subjects were aged 12 months or older.

### 3.1. Demographic Data

There were 84 (55.6%) male and 67 (44.4%) female subjects. The male to female subjects’ ratio was fairly equal for each age group. Malays formed the largest ethnic group (83.4%), followed by Indians (9.3%), Chinese (6.6%) and others (0.66%). Additionally, the mean age for mothers was 30.42 ± 4.38 (age range between 19 to 42 years). The socio-demographic characteristics of the subjects are shown in Table 2.

The majority of the mothers (54.3%) had secondary school level education, working in the private sector (37.7%) and were non-smokers (92.7%). The majority had a monthly household income between RM1500 and RM3499 (33.8%). Some infants and young children had either one parent having myopia (39.7%). Most of the fathers (56.3%) were smokers.

### 3.2. Distribution of Refractive Error

The mean spherical equivalent (SE) ± standard deviation (SD) for the right eye (RE) and left eye (LE) was +0.85 ± 0.97 D and +0.86 ± 0.98 D, respectively. The median SE ± interquartile range (IQR) for RE and LE was +0.75 ± 1.38 D and +0.88 ± 1.25 D, respectively. Since there was a strong correlation between SE of RE and LE (*r* = 0.905, *n* = 151, *p* = 0.000), only the RE results were used for subsequent analysis. The Mann Whitney U test showed that there was no significant difference of median SE between male (mean +0.78 D ± 0.94 and median +0.63 D ± 1.44) and female (mean +0.93 D ± 1.00 and median +0.88 D ± 1.00); (*Z* = −0.72, *p* = 0.48). The median of spherical equivalent between ethnicity was found to be significantly different (*p* < 0.05). Based on post hoc analysis and Bonferroni correction, Malay ethnicity had significantly higher median SE (0.88, IQR 1.25) compared to Chinese (0.13, IQR 1.63).

The mean SE differed significantly across the five age groups (*p* < 0.001). Bonferroni correction post-hoc test indicated that 6 to 11.9 months age group showed significantly higher median SE (+1.50, IQR 1.31) compared to 12 to 17.9 months (+0.88, IQR 1.50; *p* < 0.05), 18 to 23.9 months (+0.63, IQR 0.91; *p* < 0.001), 24 to 29.9 months (0.50, IQR 1.25; *p* < 0.001) and 30 to 36 months (0.63, IQR 1.25; *p* < 0.001). The mean and median SE was higher in 6 to 11.9 months age group and later gradually decreased with age.

The mean cylindrical power ± standard deviation (SD) for RE and LE were −0.99 D (± 0.60) and −1.01 (± 0.66), respectively. The median cylindrical power ± IQR for RE and LE were −1.00 (IQR 1.00), and −0.75 D (IQR 1.00), respectively. Even though not statistically significant (*p* = 0.14), the median cylindrical power decreased with increasing age. The distribution of refractive error is shown in Table 3.

The overall prevalence of refractive error is presented in Table 4. Prevalence of myopia, hyperopia, astigmatism and anisometropia were 1.3%, 12.6 %, 26% and 0.7%, respectively. Astigmatism (≥ −1.50 DC) was the most common type of refractive error among the study population. It can be seen that 39.4% (*n* = 17) of astigmatism was found in 6 to 11.9 months age group and the highest (30.0%, *n* = 3) was in Chinese. Hyperopia (≥ +2.00 D) was the second most common type of refractive error. The highest percentage of hyperopia was found in Indian population (28.6%, *n* = 4), followed by Malay (11.9%, *n* = 15) and none in Chinese. Similar to the prevalence of astigmatism, most of the hyperopia was also found in 6 to 11.9 months age group. It was also apparent that both astigmatism and hyperopia decreased with increasing age. Myopia (≥ −1.00 D) was found in 1.3% of the population with older age, that is, 24 to 36 months old. 

### 3.3. Association of Refractive Error Group with Gender and Race.

**Figure 1:**
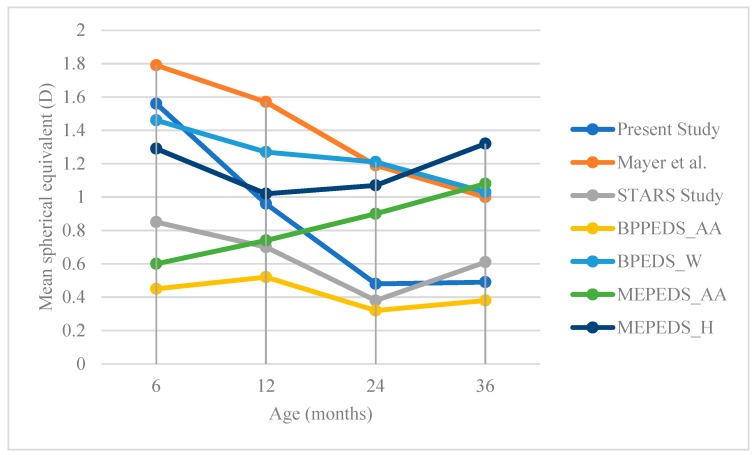
Change of mean spherical equivalent with age of different studies reported by Mayer et al. (2001) [20], STAR Study (Dirani et al. 2001) [4], BPEDS: Baltimore Pediatric Eye Disease Study (2009); MEPEDS: Multi-Ethnic Pediatric Eye Disease Study (2010). AA: African American; W: White; H: Hispanic.

There was no significant association between refractive error group (≥ +2.00 or ≥ −1.00 D) to race (*p* = 0.23) and gender (*p* = 0.88). The results are shown in Table 5. Refractive error (SE) and age were significantly correlated, rs (149) = −0.41, *p* < 0.001. (Figure 1)

## 4. Discussion

The present study was a pilot conducted to explore the refractive error status in a sample of healthy infants and young children in one health clinic in Sentul aged 36 months and less because studies on the refractive error in this population were limited, particularly in Malaysia. A small sample size of 151 infants and young children were examined. As the sample size was small, the results are the only representative of this population in the Sentul Health Clinic and cannot be generalized for the whole infant and young children population in Malaysia.

Astigmatism was the most common type of refractive error found in this study population with the prevalence rate of 25.8%, followed by hyperopia, myopia and anisometropia, with 12.6%, 1.3% and 0.7%, respectively. Our findings are in agreement with the results of Mayer et al. [20] who found the prevalence of astigmatism (defined as ≥ −1.00 D) to be 25% in their population. However, other studies [4,20] showed a lower prevalence of astigmatism even though the definition of astigmatism was similar to our present study. In the Multi-Ethnic Pediatric Eye Disease Study [21], the prevalence of astigmatism was 16.8% in Hispanic and 12.7% in African Americans, whereas Dirani et al. [4] found the prevalence of astigmatism to be 8.6% in young Singaporean children. The higher prevalence of astigmatism that was found in this study, as compared to Dirani et al. [4] and Multi-Ethnic Pediatric Eye Disease Study [21] is not unexpected because of the age differences between the two studies. The age group in our study was younger (6 to 36 months) as compared to Multi-Ethnic Pediatric Eye Disease Study [21] and Dirani et al. [4], with wider age range (6 to 72 months). Majority of astigmatism in this study was found within 6 to 11.9 months age group, and the prevalence decreased with age. This result was similar to other studies [17,22,23] who also found that astigmatism declined with age. As age increased, changes in lid pressure can produce changes in the amount of astigmatism.

Hyperopia (≥ +2.00) was the second most prevalent refractive error in our study (12.6%). The result was lower compared to Multi-Ethnic Pediatric Eye Disease Study [5] which showed a higher prevalence of hyperopia (defined as ≥ +2.00) in Hispanic than African-American children aged between 6–72 months (26.9% versus 20.8%, *p* < 0.001, respectively). Giordano et al. [24] who defined hyperopia as ≥ +3.00, found hyperopia to be higher in white children (8.9%), but slightly lower in African-American children (4.4%) aged between 6–72 months. Kleinstein et al. [13] showed that whites had the largest prevalence of hyperopia (defined as ≥ +1.25) at 19.3%, followed by Hispanic, 12.7%; African American, 6.4%; and Asian, 6.3%. However, the study was in an older age group (5 to 17 years), and the definition of hyperopia was different from ours.

The prevalence of myopia (≤ −1.00) was 1.3% in our study. The prevalence was higher in other studies [4,5,23,24] compared to ours. This could be due to a difference in the definition of myopia and age group.

Anisometropia was rare in our sample (0.7%), which was similar to the studies of Mayer et al. [20] and Dirani et al. [4]. Although anisometropia is uncommon, it is important to detect anisometropia, because if left uncorrected through infancy, is likely to lead to the development of amblyopia. The variation of the types of refractive errors found in all these studies could be related to the differences in research protocols, as well as ethnicity, environment factors and genetic make-up of the different population.

Figure 1 illustrates the comparison of changes of mean spherical equivalent with age, of different studies. At six months age, all studies showed the presence of hyperopia, with mean SE between +0.45 to +1.79 D. In the present study, there was a decrease in SE with age. The trend of decrease in SE with age following the normal process of emmetropization in our study was almost similar to Mayer et al. [20] and Dirani et al. [4]. On the contrary, two studies [5,24] did not find the trend towards emmetropia in either African American and White or African American and Hispanic, respectively. The increase of mean SE towards greater hyperopia in those two population-based studies may explain why there is more hyperopia in western children than in Asian children, who reported more myopia [13].

The mean SE in our study was less hyperopic compared to the study by Mayer et al. [20], but more hyperopic compared to other studies. The mean SE reported by Dirani et al. [4] in Singaporean Chinese Children is lower compared to the other two major population-based studies. This seems to indicate that the children from Chinese ethnicity may start with lower SE; hence, they may have a higher prevalence of myopia at school-age. Prevalence of myopia has been reported to be high in Chinese ethnicity school children, for example in Hong Kong is 36.7% [25], in Singapore is 36.3% [26] and in Shunyi, China is between 36% to 43% [27].

Presence of refractive error in infants and young children was significantly associated with age. Children in 6 to 11.9 months age group was found to have a higher proportion of refractive error compared to the older age group. This finding is in agreement with Ingram and Barr [17], Mayer et al. [20] and Mutti et al. [28], and could be explained by the process of emmetropization. This illustrates the findings of high refractive error at a very young age may be normal. However, it is important to observe the trend of refractive error change in these children as some studies [17,18] have shown that there is a higher risk of amblyopia at a later age if the child has > +3.50 D at one-year-old.

## 5. Conclusions

Astigmatism (≥1.50 DC) was the most common type of refractive error found in this study population with a prevalence rate of 25.8%, followed by hyperopia, myopia and anisometropia, with prevalence rates of 12.6%, 1.3% and 0.7%, respectively. Presence of refractive error was significantly associated with age only. Children in the younger age group were found to have higher amounts of astigmatism and hyperopia compared to the older age group. There was a significant reduction in hyperopic refractive error towards emmetropia with increasing age. However, in this pilot study, the 95% confidence intervals for the prevalence of refractive errors were very wide. Since this study only assessed refractive error in infants and young children in one health clinic, a larger study needs to be undertaken, with a wide age range before making recommendations for the policy of practice.

## Figures and Tables

**Table 1:** Estimated pool prevalence (EPP) of myopia, hyperopia and astigmatism in children by WHO regions.

WHO Regions	Astigmatism	Hyperopia	Myopia
	%EPP (95% CI)	%EPP (95% CI)	%EPP (95% CI)
South-East Asia	9.8 (6.3−13.2)	2.2 (1.2−3.3)	4.9 (1.6−8.1)
Western Pacific	12.1 (8.4−15.8)	3.1 (1.9−4.3)	18.2 (10.9−25.5)
Africa	14.2 (9.9−18.5)	3.0 (1.8−4.3)	6.2 (4.8−7.6)
Americas	27.2 (26−28.4)	14.3 (13.4−15.2)	8.4 (4.9−12)
Europe	12.9 (4.1−21.8)	9 (4.3−13.7)	14.3 (10.5−18.2)
Eastern Mediterranean	20.4 (14.5−26.3)	6.8 (4.9−8.6)	9.2 (8.1−10.4)
All	14.9 (12.7−17.1)	4.6 (3.9−5.2)	11.7 (10.5−13.0)

**Table 2:** Socio-demographic Characteristics of Infant and Young Children (*n* = 151).

Characteristics	Frequency (%) (Male:Female) (95% CI)	Mean ± SD	95% Confidence Interval	Median (Interquartile range, IQR)
**Age (months)**		18.09 (7.95)	18.8–19.4	18.00 (12.00)
**Age (months) by group**				
6–11.9	33 (21.9)			
	(21:12)			
	(15.3–28.5)			
12–17.9	39 (25.8)			
	(21:18)			
	(18.8–32.8)			
18–23.9	38 (25.2)			
	(19:19)			
	(18.3–32.1)			
24–29.9	22 (14.6)			
	(13:9)			
	(9.0–20.2)			
30–36	19 (12.6)			
	(10:9)			
	(7.3–17.9)			
**Race/ethnicity**				
Malay	126 (83.4)			
Chinese	10 (6.6)			
Indian	14 (9.3)			
Other	1 (0.7)			
**Spherical equivalent, D**		0.85 (0.97)	0.70–1.0	0.75 (1.38)
**Mothers age (years)**		30.42 (4.38)	29.7–31.1	30.00 (5.00)
**Mother’s highest education level**				
Primary school	8 (5.3)			
Secondary school	82 (54.3)			
Diploma	35 (23.2)			
Degree and above	26 (17.2)			
**Mother’s occupation**				
Housewife	53 (35.1)			
Government	32 (21.2)			
Private	57 (37.7)			
Self-employed	9 (6.0)			
**Household income (RM)**				
>5000	42 (27.8)			
3500–5000	49 (32.5)			
1500–3499	51 (33.8)			
<1500	9 (6.0)			
**Mother’s smoking history**				
None	141 (93.4)			
Current smoker	5 (3.3)			
Past smoker	5 (3.3)			
**Father’s smoking history**				
None	53 (35.1)			
Current smoker	86 (57.0)			
Past smoker	12 (7.9)			
**Parental myopia**				
None	59 (39.1)			
Mother	60 (39.7)			
Father	13 (8.6)			
Both	19 (12.6)			

**Table 3:** Mean and median refractive error by age in infants and young children.

Age, Month (*N*)	SE (D)					Cylinder (D)				
	Mean	Standard Deviation	95% Confidence Interval	Median	IQR	Mean	Standard Deviation	95% Confidence Interval	Median	IQR
6–11.9 (33)	1.56	0.92	1.23–1.89	1.50	1.31	−1.12	0.59	−1.33 to −0.91	−1.00	0.75
12–17.9 (39)	0.96	1.10	0.65–1.34	0.88	1.50	−0.99	0.61	−1.19 to −0.80	−1.00	0.75
18–23.9 (38)	0.50	0.66	0.29–0.72	0.63	0.91	−1.03	0.47	−1.18 to −0.88	−1.00	0.75
24–29.9 (22)	0.48	0.78	0.13–0.82	0.50	1.25	−0.82	0.53	−1.05 to −0.58	−0.75	1.00
30–36 (19)	0.49	0.49	0.11–0.82	0.63	1.25	−0.87	0.84	−1.28 to −0.46	−0.75	0.50

**Table 4:** The overall prevalence of refractive error by gender, age and race.

Parameters	Hyperopia	Emmetropia	Myopia	Astigmatism	Anisometropia
**Gender**					
Male (*n* = 84)	10 (11.9)	72 (85.7)	2 (2.4)	22 (26.2)	0 (0.0)
	(5.9–20.8)	(76.4–92.4)	(0.3–8.3)	(17.2–36.9)	(0.0–4.3)
Female (*n* = 67)	9 (13.4)	58 (86.6)	0 (0.0)	17 (25.4)	1 (1.5)
	(6.3–24)	(76.0–93.7)	(0.0–5.4)	(15.5–37.5)	(0.0–8.0)
**Age (months)**					
6–11.9 (*n* = 33)	11 (33.3)	22 (66.7)	0 (0.0)	17 (39.4)	0 (0.0)
	(18.0–51.8)	(48.2–82.0)	(0.00–10.6)	(22.9–57.9)	(0.0–10.6)
12–17.9 (*n* = 39)	6 (15.4)	33 (84.6)	0 (0.0)	8 (23.1)	1 (2.6)
	(5.9–30.5)	(69.5–94.1)	(0.0–9.0)	(0.11–0.39)	(0.1–13.5)
18–23.9 (*n* = 38)	1 (2.6)	37 (97.4)	0 (0.0)	8 (28.9)	0 (0.0)
	(0.1–13.8)	(86.2–99.9)	(0.0–9.3)	(15.4–45.9)	(0.0–9.3)
24–29.9 (*n* = 22)	1 (4.5)	20 (90.9)	1 (4.5)	4 (18.2)	0 (0.0)
	(0.1–22.8)	(70.8–98.9)	(0.1–22.8)	(5.2–40.3)	(0.0–15.4)
30–36 (*n* = 19)	0 (0.0)	18 (94.7)	1 (5.3)	2 (10.5)	0 (0.0)
	(0.0–17.6)	(74.0–99.9)	(0.1–0.26)	(1.3–33.1)	(0.0–17.6)
**Race**					
Malay (*n* = 126)	15 (11.9)	109 (86.5)	2 (1.6)	32 (25.4)	1 (0.8)
	(6.8–18.9)	(79.3–91.9)	(0.2–5.6)	(18.1–33.9)	(0.0–4.3)
Chinese (*n* = 10)	0 (0.0)	10 (100)	0 (0.0)	3 (30.0)	0 (0.0)
	(0.0–30.8)	(69.2–100)	(0.0–30.8)	(6.7–65.2)	(0.0–30.8)
Indian (*n* = 14)	5 (28.6)	10 (71.4)	0 (0.0)	4 (28.6)	0 (0.0)
	(8.4–58.1)	(0.42–0.92)	(0.0–23.2)	(8.4–58.1)	(0.0–23.2)
Others (*n* = 1)	0 (0.0)	1 (100)	0 (0.0)	0 (0.0)	0 (0.0)
	(0.0–97.5)	(2.5–100)	(0.0–97.5)	(0.0–97.5)	(0.0–97.5)
**Total**	19 (12.6)	130 (86.1)	2 (1.3)	39 (25.8)	1 (0.7)
	(7.7–19.0)	(79.5–91.2)	(0.2–4.7)	(19.1–33.6)	(0.0–3.6)

**Table 5:** Association of refractive error group with gender and race.

Characteristics	Cases	Number (%) of Subjects		*p*-Value
		With Refractive Error (*n* = 21)	Without Refractive Error (*n* = 130)	
**Race**				
Malay	126	17 (13.5)	109 (86.5)	0.23
		(8.1–20.7)	(79.3–91.9)	
Chinese	10	0 (0.0)	10 (100.0)	
		(0.0–30.8)	(69.2–100)	
Indian	14	4 (28.6)	10 (71.4)	
		(8.4–58.1)	(41.9–91.6)	
**Gender**				
Male	84	12 (14.3)	72 (85.7)	0.88
		(7.6–23.6)	(76.4–92.4)	
Female	67	9 (13.4)	58 (86.6)	
		(6.3–24.0)	(76.0–93.7)	
